# Unravelling the Mystery inside Cells by Using Single-Molecule Fluorescence Imaging

**DOI:** 10.3390/jimaging9090192

**Published:** 2023-09-19

**Authors:** Julian Zalejski, Jiachen Sun, Ashutosh Sharma

**Affiliations:** Department of Chemistry, University of Illinois Chicago, Chicago, IL 60607, USA; jzalej2@uic.edu (J.Z.); jsun71@uic.edu (J.S.)

**Keywords:** protein–protein interaction, medical diagnostics, cell imaging and signaling, total internal reflection fluorescence, protein dynamics

## Abstract

Live-cell imaging is a powerful technique to study the dynamics and mechanics of various biological molecules like proteins, organelles, DNA, and RNA. With the rapid evolution of optical microscopy, our understanding of how these molecules are implicated in the cells’ most critical physiological roles deepens. In this review, we focus on how spatiotemporal nanoscale live-cell imaging at the single molecule level allows for profound contributions towards new discoveries in life science. This review will start by summarizing how single-molecule tracking has been used to analyze membrane dynamics, receptor–ligand interactions, protein–protein interactions, inner- and extra-cellular transport, gene expression/transcription, and whole organelle tracking. We then move on to how current authors are trying to improve single-molecule tracking and overcome current limitations by offering new ways of labeling proteins of interest, multi-channel/color detection, improvements in time-lapse imaging, and new methods and programs to analyze the colocalization and movement of targets. We later discuss how single-molecule tracking can be a beneficial tool used for medical diagnosis. Finally, we wrap up with the limitations and future perspectives of single-molecule tracking and total internal reflection microscopy.

## 1. Introduction

Single-molecule imaging is a widely used fluorescence microscopy technique that tracks the movements of various individual molecules through the detection of an attached fluorescence molecule. This process allows for determining the behavior and dynamics of individual molecules within living cells [1,2,3]. It allows researchers to gain insights into various cellular processes, including protein–protein interactions (PPIs), membrane dynamics, intracellular transport, and signaling pathways [4,5,6,7,8,9]. Single-molecule tracking (SMT) typically involves the use of fluorescently labeled molecules, such as proteins or nucleic acids, which can be visualized and tracked using fluorescence microscopy [1,10]. These fluorescent labels are developed to emit light when excited by a specific wavelength, allowing researchers to detect and follow individual molecules within a cell [11,12]. Traditionally, live-cell imaging involves a compromise between obtaining image quality and maintaining the health of the cell. This is performed by using a strong enough powered laser to excite the fluorophore without harming the live cell over the course of the experiment, and as a result this balancing act can lead to limited spatial and temporal resolutions [13]. Typical live-cell imaging techniques include confocal microscopy, epifluorescence/traditional fluorescence microscopy, and total internal reflection fluorescence microscopy (TIRFM). While both have a long history of capturing cellular dynamics with excellent resolution for several decades, they are still popular and powerful technologies in academic and industrial research. Concurrently, the modern design of super-resolution microscopy (SRM) techniques shines new insights into single-molecule imaging in live cells, with the three popular SRM being structured illumination microscopy (SIM); stimulated emission depletion microscopy (STED); and single-molecule localization microscopy (SMLM) [14]. All of which allow resolution beyond the diffraction limit of light of 250–300 nm [15] and can visualize biological structures in a detailed manner, more precisely, 5–10 nm [14]. However, being a super powerful tool, confocal imaging does carry limitations that could make it difficult to perform SMT [16,17]. As of right now, this means that the most common form of single-molecule detection is executed with TIRF.

Conventional fluorescence microscopes like confocal use a vertical laser beam to excite the sample, leaving the excitation volume in the z-axis direction very large. TIRF microscopy is the opposite; the excitation laser is angled in such a way that it completely reflects off the glass sample slide with no light penetrating the sample. While the evanescent wave oscillates with the same frequency as the reflected light, it still excites any fluorescent molecules within this small area, typically a ~100 nm optical section from the bottom of the sample [18]. Thus, it ensures a high signal-to-noise ratio and reduces background interference [19,20]. Meanwhile, it is also much less phototoxic to the live sample, which could allow for a longer monitoring time. Thus, TIRFM is well suited for studying cellular signaling and vesicle trafficking research, such as the kinetics of membrane proteins or protein–membrane interactions.

**Figure 1:**
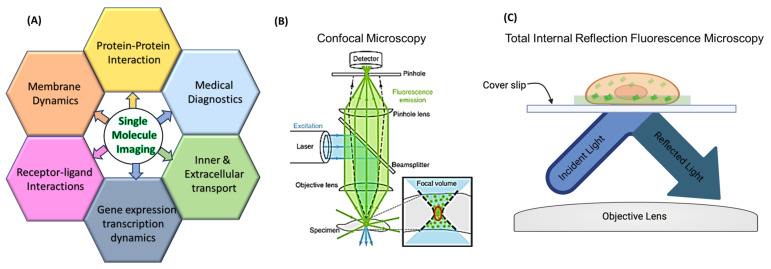
(**A**) Applications of single-molecule imaging in drug discovery. (**B**) Schematic representation of confocal microscopy (**C**) Schematic representation of total internal reflection fluorescence microscopy.

In most cases, illness, ailments, and diseases can be boiled down to disruptions in cellular behaviors. Particularly, changes in the way proteins interact with each other, proteins interact with lipids [21], or organelle dynamics [22,23]. Currently, diagnosis of illness comes from characterizing the changes in protein dynamics that give rise to behavioral changes like proliferation and metastasis in cancer cells [24,25], and a physical change in cell shape like in sickle cell anemia [26]. It is difficult to diagnose a particular illness or disease whose symptoms can overlap with one another but be caused by different sources [27,28,29]. This is where SMT could become beneficial to better diagnose illnesses and diseases, lowering the chances of misdiagnosis. By looking into afflicted cells, it could be possible to pinpoint which proteins have changed behavioral patterns, causing symptoms for the patient, and create a diagnosis from there. SMT can not only be beneficial for diagnostic purposes, but it can also further expand fields in drug delivery, mechanisms of pathogen invasion, and other possibilities explained further in this review (Figure 1).

In this review, we start with a brief overview of current uses of SMT, later expanding on recent SMT publications that contribute to new approaches that make SMT a more robust technique, tracking cellular organelles and their interactions with proteins and each other, and concluding with how SMT can be utilized for medical diagnosis.

## 2. Brief Overview

In recent years, the rapid development of SMLM technology has made it possible to accurately observe and manipulate dynamic processes such as position, orientation, distribution, and chemical reactions of individual biomolecules at the nanometer scale [30,31,32,33,34]. SMT is a powerful technique used in cell biology and biophysics to study the behavior and dynamics of individual molecules within live cells. Thanks to recent advancements in optics, TIRF microscopy has become the go-to method for tracking complex biological interactions in real-time [35]. Thanks to its ability to excite fluorescent proteins only about 100 nm away from the coverslip, reducing out-of-focus background florescence, users can expect a high signal-to-noise ratio [35]. TIRFM has been implemented in many different experiments, tracking a whole host of protein interactions and organelle formation and dynamics.

SMT in live cells by fluorescence would not be possible if it was not for Iino et al., 2001. Being the first to successfully image single green fluorescent proteins (GFP) inside a living cell, Iino et al., 2001 published their findings showing for the first time that E-cadherin is found to be oligomers of various sizes on the free cell surface [36]. With the ability to image fluorescent proteins inside a living cell, SMT by fluorescence quickly gained traction in the biology/chemistry fields. Early applications of TIRFM for SMT were used to track receptor–ligand interactions on the plasma membrane [37]. Using fluorescently tagged EGF (epithelial growth factor) molecules, Sako et al., 2000 have shown that epithelial growth factor receptors (EGFRs) dimerize before the binding of second EGF molecules [37]. This mechanism of EGFR activation was then confirmed using single-molecule fluorescence-resonance energy transfer (FRET) [37]. In just a few years, SMT has become a very dynamic technique that can serve as a tool for more than just tracking transmembrane receptors. SMT has been implemented to track how transcription factors search for and assemble at their DNA target sites [38]. The authors continually set out to improve and expand SMT to make it a more robust and multi-applicable technique to extract as much information from a cell as they never thought would be possible just a few years prior. Moving away from TIRFM, published SMT can be performed with the combination of light sheet microscopy and high-speed imaging to afford a very sensitive-molecule tracking technique [39]. This approach to SMT makes it possible to track molecules in solution, living cells, and even tissue [39]. These current approaches are a far cry from where it all started. This section will expand on the current uses of SMT for unraveling mysteries inside a cell, from membrane dynamics, PPIs, intercellular transport, and more.

## 3. Membrane Dynamics

The cellular membrane is one of the most dynamic and integral parts of the cell. Not only does it keep the cell together, but it also plays a crucial role in signal transduction, cell-to-cell signaling, cellular uptake, and much more. SMT allows researchers to study the dynamics of membrane proteins and lipids in live cells, and by tracking individual molecules, scientists can investigate the diffusion, clustering, and interactions of proteins within and around the cell membrane. Investigating membrane dynamics can help in understanding membrane organization, protein trafficking, cellular structures, and signaling processes.

**Figure 2:**
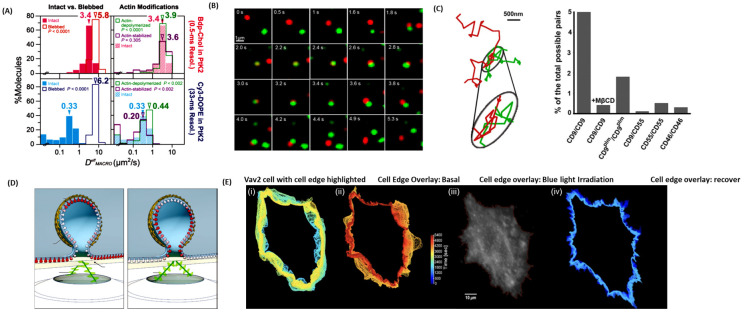
(**A**) In the PtK2-cell PM, Bdp-Chol diffused 10× faster than Cy3-DOPE in the intact PM, whereas they diffused at about the same rate in the blebbed PM [40]. (**B**) Time-lapse Imaging showed simultaneous single-molecule tracking of two differentially labeled CD9 molecules with a Fab fragment conjugated with Atto647N (red) or with Cy3B (green) (**B**) Representative trajectory of CD9 dynamic colocalization, adapted from [43]. (**C**) Quantitative analysis of single-molecule colocalization. Under these conditions, dynamic CD9 colocalization was observed in 5% of the total possible pairs [43]. (**D**) Polarized-TIRF microscopy permits imaging of membrane bending at clathrin-coated structures. Schematic representation of pol-TIRF. DiI–C18 orients its dipole moment with the plasma membrane. S-polarized TIRF illuminates horizontal dye molecules, whereas P-polarized TIRF selectively excites vertical dye molecules. The P/S provides contrast for membrane bending, adapted from [45]. (**E**) Tracking cell edges, adapted from [47]: (**i**) HeLa cells with the cell edge highlighted before, during, and after opto-genetically stimulated VAV2 release. (**ii**) traces of the cell edge overlaid at 10 s intervals before optogenetic stimulation. (**iii**) traces overlaid during VAV2 release, (**iv**) traces overlaid after blue light irradiation had been halted; Warmer colors indicate later time points.

Cholesterol plays a key role in plasma membrane organization [40], and with a recent publication shedding light on the asymmetric trans-bilayer distribution of cholesterol on the plasma membrane, this difference in concentration between the two layers could be important to various signaling pathways inside the cell [41]. What is poorly understood is how cholesterol concentration is regulated in the plasma membrane. In the research published by Hiramoto-Yamaki et al., 2014 authors try to uncover what allows cholesterol to flip-flop between the outer plasma membrane and the inner plasma membrane. With the development of a bodipy488-conjugated cholesterol molecule (Bdp-Chol) and a Cy3-conjugated dioleoylphosphatidylethanolamine (Cy3-DOPE), authors were able to study the behavior of these two lipids in the plasma membrane using single fluorescent-molecule imaging [40]. Where the cholesterol was able to flip-flop between the plasma membranes at a relatively quick pace, the Cy3-DOPE took about ~10× slower than Bdp-Chol40 (Figure 2A). The authors attributed this difference in flip-flop speed to the actin-based membrane skeleton, which has little effect on cholesterol flipping and makes it very difficult for other phosphatidylinositol lipids to flip-flop [40].

Tetraspanins are molecular scaffolding proteins found in the plasma membrane that distribute membrane proteins into highly organized microdomains [42]. Tetraspanins regulate specific proteins and their functions, for example, adhesion-mediated (integrins/FAK), receptor-mediated (EGFR, TNF-α, c-Met, c-Kit), and intracellular signaling (PKC, PI4K, β-catenin) proteins [42]. Composed of four transmembrane domains, it is believed that interactions with these domains and cell surface proteins allow for the formation of a “tetraspanin web”, uniquely segregating proteins who associate with tetraspanins, similar to the proposed lipid raft hypothesis for lipids [43]. However, evidence of live-cell sequestration of associated proteins by tetraspanins was missing, leaving a hole in the proposed hypothesis. By utilizing single-molecule fluorescence microscopy with TIRFM, it was shown that these tetraspanin micro-domains are transient and highly dynamic, with two separate modes of interaction being identified. The first form of interaction leading to tetraspanin web formation is independent of either cholesterol or cytoskeletal interactions with the tetraspanin, where the strength of these tetraspanin pools is directly related to protein–protein interactions [43]. It was also noticed that these tetraspanin-enriched areas were not fully connected with the rest of the membrane but were in permanent exchange with it [43]. The second mode of action leading to tetraspanin-web formation relies heavily on protein–lipid interactions [43]. By co-diffusion of two tetraspanin proteins that are outside of the tetraspanin-enriched areas but are interacting with other protein partners or even lipids, the tetraspanin web can form [43] (Figure 2B). The data suggested single tetraspanin clusters interact by exchange of CD9 molecules (Figure 2C), but this interaction is dependent on cholesterol and palmitoylation, and the precise role of how these lipids mediate the interaction remains unclear. This new approach to monitoring tetraspanin uncovered a dynamic web of protein–protein–lipid interactions that updates the current narrative that these tetraspanins interact similarly to raft microdomains.

Cellular endocytosis generates small membrane vesicles that envelope and transport a wide range of molecules from the plasma membrane into the cytoplasm through a process known as clathrin-coated endocytosis [44]. It is believed that clathrin plays a pivotal role in shaping the plasma membrane into an endosome and recruiting other proteins that aid in the formation of endosomes, like scission proteins and actin [44,45]. Over 35 years ago, electron microscopy showed clathrin oligomerizes into a flat hexagonal lattice on the plasma membrane, where the lattice then rearranges into a pentagonal formation when the endosome is being formed [45]. With major pushback from the science community due to the energy cost of transitioning from one lattice formation to another, there is major uncertainty in the current fundamental mechanism. Scott et al., 2018 set out to capture the dynamics of membrane topographical changes during endocytosis by using pol-TIRFM [45] (Figure 2D). Developed by Axelrod and colleagues, polarized-TIRFM could generate contrasts between vertical and horizontal changes/movements in plasma membranes in living cells [45,46]. Expression of fluorescent protein-tagged clathrin and dynamin showed two modes of action. First, clathrin-coated structures form curved membranes and recruit clathrin as the depression is forming; second, at the plasma membrane, clathrin is recruited from large sheet-like structures that later bend to form pits. Since both behaviors were detected in living cells, this led to the suggestion that clathrin-mediated endocytosis is a flexible process and that local biophysical factors play a large part in determining what mode of endocytosis is used [45].

Arguably, the cell plasma membrane is one of the most complex aspects of the cell. As discussed above, it mediates signal transduction pathways, regulates plasma membrane proteins and lipids, and even naturally oscillates at a frequency of 3 mHz [47]. An optogenetic approach was used to control protein position repeatedly and reversibly within a cell to monitor the movement of the cellular membrane when sequestering and releasing proteins associated with membrane dynamics [47]. LOVTRAP (LOV2 trap and release of protein) is made of two parts: Zdk, which binds selectively to the dark state of LOV2, and LOV2 and the photosensor domain [47]. By anchoring either Zdk or LOV2 away from the action site of the protein of interest (POI) and the POI to the subsequent other half (nonanchored half), the POI would be sequestered in the dark. When irradiated with light, Zdk would dissociate from LOV2, leaving the POI to travel to the site of action [47]. Fusing Vav2 (a protein known to regulate cellular protrusion) with the LOVTRAP system shows very striking results in terms of membrane behavior [47] (Figure 2E). Irritating cells expressing LOVTRAP-Vav2 complexes with 50 s “on” and 250 s “off” cycles generated highly synchronous protrusion–retraction cycles with a 3.3 mHz frequency [47].

## 4. Receptor–Ligand Interactions

Single-molecule tracking enables the observation and quantification of interactions between cell surface receptors and their ligands. By monitoring the binding and unbinding events at the single-molecule level, researchers can gain insights into the kinetics, affinity, and spatial distribution of these interactions. This information is crucial for understanding cell signaling pathways and receptor-mediated processes.

**Figure 3:**
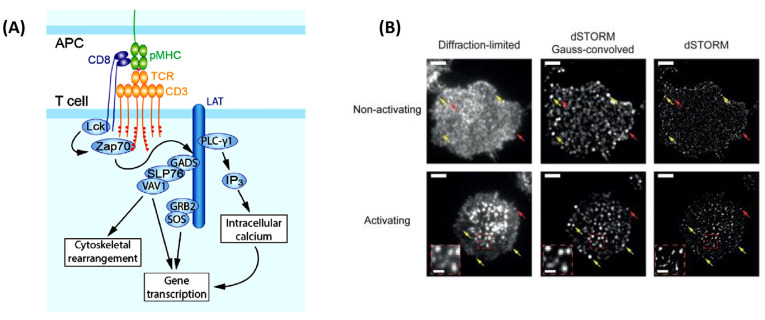
(**A**) Binding of TCR and CD8 (or CD4) triggers the proximal signaling that involves phosphorylation of CD3 ITAMs by Lck, ITAM binding and activation of Zap70, and phosphorylation of LAT. Recruitment of multiple adaptor and signaling molecules to LAT and their activation further propagate and direct downstream signaling for various cellular functions, adapted from [48]. (**B**) Diffraction-limited images, adapted from [49], dSTORM localization maps, and back-calculated diffraction-limited images based on dSTORM localization maps (center) of fixed primary murine CD4+ TEFF cells labeled with H57-AF647 Yellow arrows: features in the dSTORM and reconstructed images with no correspondence in the original diffraction-limited image. Red arrows: features that do have such a correspondence. Inserts (red dashed boxes) show zooms of regions in activated cells with pronounced microclustering, where high localization densities are clearly correlated with high molecular densities.

This is no truer than understanding how T-cells become activated. With the explosion of immunotherapies to treat cancer, a complete picture of T-cell activation is necessary before their implementation as therapeutics. SMT can help elucidate how T-cells become activated in response to the direct interaction of the T-cell antigen receptor (TCR) and its conjugate peptide presented on the antigen-presenting cell (APC) surface [48] (Figure 3A). This was soon visualized using TIRFM, where the images uncovered a whole host of changes in protein organization during the formation of an immunological synapse (leading to T-cell activation) between T-cells and a functionalized lipid bilayer [48]. This rearmament leads to the clustering of the central super-molecular activation cluster (cSMAC), the peripheral super-molecular activation cluster (pSMAC), and the distal super-molecular activation cluster (dSMAC) to form a pseudo “bulls eye pattern” around the immunological synapse [48]. Researchers’ identification of what happens inside the T-cell during activation and TCR clustering have posed a point of contention in the immunology field [49]. Previous studies on Jurkat and primary T-cells have concluded nanoclustering of TCRs is opposed to a diffuse arrangement of TCRs around the cell membrane before the activation of these cells [49]. It was thought that this nanoclustering was necessary for achieving the antigen sensitivity and specificity that are observed in TCRs [4]. However, Rossboth et al., 2018 have shown through their method of label-density-variation signal molecule localization microscopy a different narrative (Figure 3B). Their method is based on deliberately varying the concertation of fluorophores to label the TCRs, affording a different way to analyze the special distribution of TCRs from previous methods [49]. Single-molecule localization microscopy has shown nanoclustering of the TCRs only exists once the T-cell is activated [49]. When the T-cell is not activated, the TCRs hold a consistently randomized distribution pattern on the plasma membrane, which is best suited for rapid antigen screening.

## 5. Protein–Protein Interactions

SMT allows the study of PPIs in real-time and at the single-molecule level. By labeling different proteins of interest and tracking their movements within cells, researchers can examine their association, dissociation, and complex formation. This provides valuable information about protein dynamics, stoichiometry, and the influence of cellular conditions on protein interactions.

**Figure 4:**
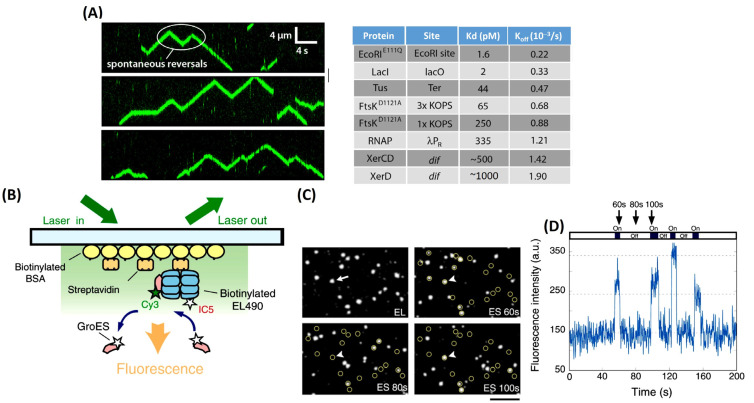
(**A**) Examples of kymographs highlighting typical examples of QD-tagged FtsKabg (shown in green) translocating on unlabeled DNA substrates; the DNA is unlabeled because intercalating dyes such as YOYO1 inhibit the translocation of FtsK. Table showed the list of roadblock proteins, indicating the experimentally determined Kd and koff values based on bulk biochemical DNA-binding measurements, adapted from [50]. (**B**) Schematic drawing of the experiment. Biotin-, IC5-labeled GroEL mutant (IC5-EL) was immobilized on the glass surface through a biotinylated bovine serum albumin–streptavidin linker. The flow cell containing the immobilized GroEL was filled with Cy3-labeled GroES (Cy3-ES), ATP, reduced lactalbumin, and the oxygen scavenger system. The association and dissociation of Cy3-ES molecules with GroEL were visualized by TIRFM, adapted from [52]. (**C**) Fluorescence images of single GroEL and GroES molecules, adapted from [52]. EL, immobilized IC5-EL. ES, Cy3-ES. The positions of IC5-EL are indicated by circles colored yellow. Arrowheads mark the positions of the IC5-EL indicated by the arrow in the first panel. Cy3-ES molecules observed outside of circles were those attached to GroEL molecules that were not fluorescently labeled. (**D**) Time course of fluorescence intensity from Cy3-ES associating and dissociating with the single GroEL molecule indicated by the arrow in (**C**). The vertical arrows in (**D**) indicate the time when the fluorescence micrographs in (**C**) were taken.

With about 55% of the dry weight of a cell coming from proteins, it would be hard to assume that proteins would not interact with one another. On the contrary, PPIs are key in signal transduction pathways as well as being integral to other cellular mechanisms. Using single-molecule imaging, the bacterial protein FtsK was visualized in real time as it collided with other proteins [50] (Figure 4A). FtsK is a bacterial translocase that is involved in bacterial chromosome dimer resolution [50,51]. During cell division, FtsK travels along the chromosome, pushing off most DNA-bound proteins to allow for continued translocation [50]. FtsK is able to move across the chromosome by harnessing the chemical energy from nucleotide hydrolysis. The chromatin is littered with many DNA-binding proteins like polymerases, helicases, and others that need to be removed before the cellular division is complete due to these proteins becoming a major source of replication fork stalling [50]. With a very crowded environment for the FtsK protein to be working in, Lee et al., 2014 decided to use single-molecule imaging to track FtsK since there is little mechanistic information regarding what happens to FtsK when it encounters an obstacle on the chromatin that it cannot push off. FtsK can push, evict, and bypass (“jump” the obstacle or reverse direction on the chromatin) almost all DNA-bound proteins, with the primary factor dictating what would happen in a collision being the relative affinity to their specific binding sites [50]. This means that the stronger the hold the protein has on the chromatin, the tougher it is for FtsK to push it off [50]. Also shown was a way to inhibit FtsK from removing XerCD by interacting with XerD [50]. The data also explain that FtsK is not the strongest motor protein and gets overpowered by RecBCD. When they collide on the same DNA molecule, it kicks an odd FtsK, indicating that RecBCD exerts a much greater force when translocating along the DNA [50].

One of the many roles’ proteins play, other than facilitating cell division, is assisting other proteins in folding through a chaperone-mediated process. GroEL and its partner GroES are the best-characterized chaperonins from *E. coil* [52]. They function together as a complex where the doubletoroid GroEL tetradecamer encapsulates non-native proteins and GroES caps the housed non-native protein in the presence of ATP [52] (Figure 4B). With the help of single-molecule tracking and TIRM, researchers are able to determine the full kinetics of the GroEL and GroES complexes [52]. By immobilizing a fluorescently labeled GroEL on a glass surface through a biotin-streptavidin linker and adding a differently fluorescently labeled GroES into the cell, one can track the process by which the GroES and GroEL form their complex [52] (Figure 4C). After the addition of GroES in the presence of ATP, signals from GroES and GroEL start to overlap, indicating GroES-GroEL binding. The complex starts to decay after 8–15 s, starting with the dissociation of GroES, which triggers the release of the trapped protein inside the GroEL cavity. The current understanding of the dissociation pattern was thought to be one step, but after signal-molecule analysis, it showed the release of GroES from GroEL occurred after a ~5 sec lag period [52] (Figure 4D). This indicated that the dissociation does not happen in one step but in two sequential transitions [52]. Through the use of signal molecule tracking and TERM, Taguchi et al., 2001 show that it is possible to determine PPI kinetics.

## 6. Inner and Extracellular Transport

Single-molecule tracking can be used to investigate the transport and trafficking of molecules within the cell. By tracking individual cargo molecules or motor proteins, researchers can study their movement along cytoskeletal tracks, such as microtubules or actin filaments. This helps in understanding intracellular transport mechanisms, organelle dynamics, and the regulation of cellular processes like vesicle trafficking.

Extracellular vesicles (EVs) are expressed by most cell types; however, due to a lack of a model organism to study the expression, movement, uptake, and fate of these vesicles, the implications of these vesicles in terms of cellular health remain elusive [53]. A protocol was developed to track BODIPY C12-labeled VLDLs’ from a zebrafish embryo yolk sack, and utilizing live fluorescence imaging with the embryos immobilized in agar allowed for the study of the biogenesis, transfer, uptake, and fate of individual endogenous single EVs from the yolk to the other parts of the fish [53]. Diving deeper into the endocytosis mechanism, by attaching a HaloTag to a platelet-derived growth factor receptor (PDGFR) and expressing the construct in Martin–Darby canine kidney cells, the movement of the growth factor being endocytosed can be accurately tracked [54]. By associating the HaloTag with a quantum dot, the endocytosis mechanism was visualized through spinning-disk confocal microscopy [54]. Live-cell imaging revealed that the HaloTag entered the cells, where the protein moved along microfilaments and microtubules to the lysosomes around the nucleus [54]. From there, the endocytosed proteins followed one of two paths. The first path is the recycling of the PDGFR by circulating endosomes around the nucellus back to the plasma membrane [54]. The second path includes full endocytosis, leading to degradation. This whole mechanism was halted by the introduction of a clathrin inhibitor, indicating that endocytosis of PDGFR is regulated by clathrin [54].

## 7. Gene Expression and Transcription Dynamics

Single-molecule tracking has been instrumental in studying gene expression and transcriptional regulation. By labeling RNA molecules or specific components of the transcription machinery, researchers can track their localization, movement, and interactions during transcriptional processes. This provides insights into RNA dynamics, transcription kinetics, and the spatiotemporal regulation of gene expression.

The functions of RNAs are just now being elucidated with the implementation of live-cell imaging. The function of RNAs has been associated with their unique subcellular localizations; however, effective live-cell imaging techniques for localization left a big hole in understanding the dynamic roles RNA played. Recently, CRISPR-dCas13 has been identified as an RNA-guided and RNA-targeting RNase protein [55]. Since it is believed that RNA dynamics indicate RNA function, visualizing in real time how RNA moves throughout the cell under different cellular conditions is key to understanding its function [55]. Screening the family of CRISPR-dCas13 proteins, dPspCas13b and dPguCas13b have been identified for direct visualization of NEAT1, MUC4, GCN4, and SatIII transcripts. These dCas13 proteins, when fluorescently tagged, have been shown to be robust, rapid, and efficient at labeling the targeted transcripts and can be visualized for quite some time [55]. These dCas13 proteins can also be combined with either MS2-MCP or orthogonal dCas13 to achieve dual-color labeling of RNAs in cells [55]. dCas13 can also be combined with dCas9 for simultaneous visualization of genomic DNA and RNA transcripts [55].

Yang et al., 2019 claimed that single-molecule fluorescence in situ hybridization (smFISH) was not suitable for live cells, but for fixed samples, live-cell fluorescence in situ hybridization (LiveFISH) has been developed for live-cell imaging. Similar to the experiments conducted by Yang et al., 2019, but using both dCas9 and dCas13 with an associated fluorescent gRNA, we were able to live track a whole host of DNA and RNA processes [56]. LiveFISH was first implemented to visualize gene editing by inducing double-stranded breaks in living cells [56]. By inducing double-stranded breaks via dCas9, expressing a fluorescently tagged, well-characterized double-strand break (DSB) sensor, and using nonactive dCas9 as a tag for the location of the DSB, the dynamics of gene-editing events were able to be tracked [56]. The DBS sensor was shown to be recruited to the locus where the break was induced (colocalization of the sensor and nonactive dCas9), and subsequently, the dissociation of the sensor was seen, which indicated successful DSB repair [56]. This method was used next to detect chromosome translocation in living cells (Haifeng Wang). Two DSBs were induced, similar to the previous experiment, and fluorescently tagged dCas9 proteins that target each break were expressed to track the chromosomes [56]. Once the breaks were induced, it was observed that the two loci paired with each other and stayed paired, as indicated by the colocalization of the two fluorescently tagged dCas9’s [56]. This observation represented endogenous and nonhomologous chromosomal repair in live cells. With a robust method of tracking DSBs and chromosomal loci, LiveFISH was next implemented for the visualization of transcription in living cells. By utilizing both a catalytically deactivated dCas13d with fluorescent gRNAs to track RNA production and a catalytically deactivated dCas9 with fluorescent gRNAs targeting LacO DNA, we were able to visualize in real time the progression of transcription [56]. When transcription was induced, the intensity of the dCas13 fluorescent gRNA increased over time, which indicated an increase in RNA production [56].

## 8. Tracking of Whole Organelles

**Figure 5:**
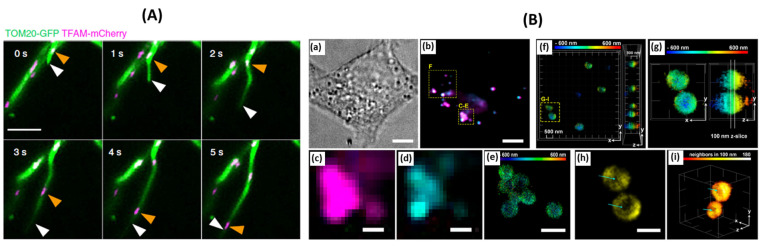
(**A**) Representative time-lapse images of MDT in a Cos-7 cell expressing Tom20-GFP and TFAM-mCherry demonstrate unsynchronized motility of TFAM-labeled nucleoids and the MDT process. White arrowheads mark the tips of tubules generated by the MDT processes. Orange arrowheads indicate the sites of nucleoids, adaped from [59]. (**B**) 3D-SMLM of lipid droplets in fixed HeLa cells, adapted from [63]. (**a**) Bright-field image of a HeLa cell. (**b**) Diffraction-limited (HILO illumination) images of droplets stained with BODIPY 493/503 and compound 6 after photoactivation. (**c**) Diffraction-limited image of lipid droplets using compound 6 after photoactivation. (**d**) Diffraction-limited image of lipid droplets using BODIPY 493/503. (**e**) 3D-SMLM image obtained with compound 6. The color bar indicates depth relative to an arbitrary focal plane. (**f**) 3D-SMLM of lipid droplets, color-coded by depth, viewed along the z and x axes. (**g**) Selected lipid droplets for 2D representation. (**h**) 2D representation of the z slice selected in panel G. This image corresponds to the x−y lateral plane. Cyan arrows indicate the absence of signals in the core of the droplet. (**i**) 3D rendering of single molecules on the phospholipid monolayer of lipid droplets. Signals are colored by density (number of neighbors within 100 nm). Cyan arrows indicate the less densely labeled core of the droplet.

Single-molecule tracking techniques have been instrumental in studying and detecting various cell organelles, providing insights into their dynamics, interactions, and functions. One organelle that has been the topic of much research is the mitochondria. The mitochondria are vital organelles involved in energy production and cell signaling [57,58]. Single-molecule tracking can be used to study mitochondrial dynamics, including their movement, fusion, and fission events. By labeling mitochondrial proteins or fluorescent probes specifically targeted to mitochondria, researchers can track individual mitochondria in living cells and investigate their distribution, interactions with other organelles, and changes in response to cellular conditions or diseases. Human mitochondrial DNA (mtDNA) encodes 13 proteins, 2 ribosomes, and 22 transfer RNAs, and mutations in any of these genes are linked to mitochondrial disfunction and neurogenerative and senescence-linked disorders [59]. This mtDNA is thought to be found throughout the inner membrane, and its separation and containment were thought to be due to a passive mechanism [59]. However, due to recent observations and experiments associated with the reduction in Drp1 levels (associated with division) and then rescuing Drp1 without rescuing mitochondrial division, a more active mechanism in mitochondrial homeostasis is thought to regulate this process [59]. Mitochondria are known to form mitochondrial dynamic tubulation (MDT), a critical mechanism that forms the mitochondrial network in the peripheral zones of mammalian cells. The formation of these protruding and retracting tubulars is driven by a motor protein called KIF5B, and a previous study has shown this protein is in close proximity to mtDNA [59]. This proximity suggests a link between the sequestering and distribution of mtDNA and the activity of KIF5B. By using multicolor live-cell super-resolution imaging, it was observed that MDT activity was high at the ER-mitochondria contact sites (EMCS), and by simultaneously tracking the movement of nucleoids, it was seen that the nucleoids traveled to the tip of the tubule (Figure 5A) [59]. The fusion of different tubules from different mitochondria leads to the transfer of nucleotides among themselves. Together, this work changes the current narrative of how mtDNA is actually actively transported, sequestered, and distributed amongst mitochondria throughout the cell, mediated by the motor protein KIF5B [59].

Well studied due to its many roles inside the cell and implication in cell stress and response to diseases, the ER is also involved in protein synthesis, folding, and lipid metabolism [60,61,62]. Single-molecule tracking can provide insights into ER dynamics and protein trafficking within the ER network. By labeling ER-resident proteins or fluorescent probes targeted to the ER, researchers can track individual ER tubules and study their movement, interactions with other organelles, and changes in response to cellular stress or disease conditions. This helps in understanding ER-related processes like protein folding, quality control, and the ER stress response. Lipid droplets not only play the role of lipid storage sites but also participate in lipophagy, signaling processes, and are associated with pathogen entry [63]. Common human diseases also related to lipid droplets include diabetes, obesity, and some viral infections. With that in mind, understanding the role lipid droplets play may afford new insights into disease progression and druggable targets. Currently, many fluorescent proteins, antibodies, and dyes are on the market to detect lipid droplets within a cell; however, very few probes exist enabling the ability to track lipid droplets and single lipid molecules in a single-molecule tracking method [63]. It has been widely published that the endoplasmic reticulum (ER) is the main site for biosynthesis and shuttling of lipids [64,65], but what was not well understood was how lipids diffused and exchanged between lipid droplets and the ER [63]. The synthesized photoactivatable palmitic probe gave the researchers the ability to image the periphery of the lipid droplet in three dimensions with a resolution beyond the limit of diffraction [63]. This probe was also able to track individual lipids being trafficked between droplets and the ER [63]. The synthesized probes were quite stable, enabling the observation of individual droplets for around 200 s, and in that time span, it was seen that around 3% of lipids leave a droplet [63]. The 3% of lipids that left the droplet did so in a few specific regions thought to be contact points between the lipid droplet and other organelles. In addition, confocal microscopy had shown the probe being shuttled in tubular structures within the cell, indicating a vast network of lipids being shuttled all over the cell. Lipid droplets bud to maintain contact with the ER, but it was not known if this budding allowed for the diffusion of lipids from the ER to the droplet or vice versa. By tagging the ER with eGFP and staining the lipid droplets with the photoactivable probe, single-molecule tracking was performed (Figure 5B) [63]. This experiment proved the authors hypothesis that indeed lipids get trafficked from the lipid droplet to the ER and back again [63]. A few molecules not associated with the droplet diffused along the ER network were also observed.

Overall, single-molecule tracking offers a unique and powerful approach to studying cellular dynamics and molecular interactions in live cells. It provides detailed information that is not achievable through traditional bulk measurements, allowing researchers to gain a deeper understanding of complex biological processes.

## 9. Medical Diagnostics

Single-molecule tracking is a powerful technique that allows researchers to monitor individual molecules in real-time. While it has various applications in different fields of science, including biology, chemistry, and physics, it also has valuable applications in medical diagnostics.

### 9.1. Protein Dynamics

As mentioned briefly, a myriad of illnesses and diseases stem from changes in protein behaviors, especially protein–protein interactions. By utilizing SMT, these changes in behaviors could be better understood to gain a full understanding of the illness on a protein level. This information could be crucial for understanding the mechanisms of various diseases and designing better targeted therapies.

Current “high throughput” methods of elucidating potential PPIs that drive diseases, especially cancers, come in the form of highly structured software analysis. In the case of determining what proteins play the most crucial role in oral cancer progression, extensive data mining techniques were used [66]. Genes associated with oral cancer were collected from PolySearch and then the genes were cross-referenced from published literature to ensure the genes and proteins PolySearch claimed to be associated with oral cancer were [66]. The catalog of genes was then cross-referenced with the STRING database for the construction of PPI networks [66]. The topology of the PPI network was extensively analyzed using software analysis, which concluded that TP53 is the central protein that plays the most critical role in oral cancer progression due to its characteristic as a bottleneck protein in the PPI network [66]. Deletion or inhibition of this protein allows the cancer to grow uncontrollably without resistance [66]. Using computational methods to find targets for cancer is extremely beneficial, if not an easier process than traditional biological processes. However, these computational methods only go so far in understanding exactly how these proteins behave in cells. This is where SMT can play a beneficial role by bridging the gap between speculation of protein behaviors via computer models and definitive answers from cells. This is especially true in the context of protein–lipid or protein–membrane dynamics.

**Figure 6:**
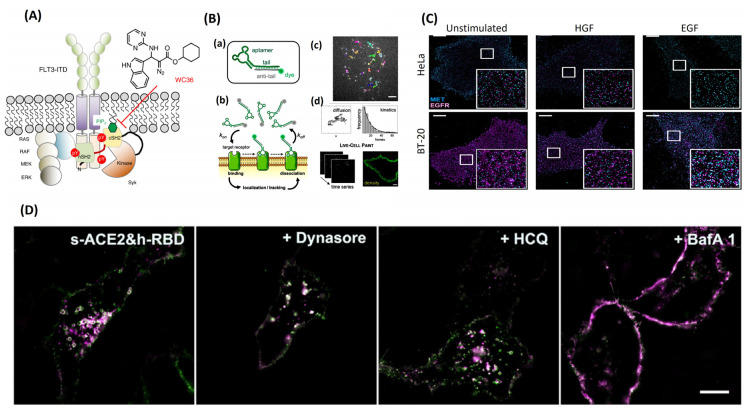
(**A**) Discovery of WC36 as a drug target for lipid–protein interaction (Syk-cSH2 and PIP3 interactions), adapted from [21]. (**B**) (**a**) The fluorescently conjugated aptamer probe. The aptamer tail (green) is annealed to its complementary anti-tail (grey), bearing a fluorophore (green sphere). (**b**) Schematic of live-cell imaging using aptamers. Thanks to TIR illumination, only aptamers (green fluorophores) that bind cell-surface receptors are selectively excited and detected. In contrast, freely diffusing aptamers (grey fluorophores) are not observed. Transient binding of aptamers to target receptors enables single-molecule imaging in nearly unperturbed living cells. (**c**) Single-molecule tracking was performed using sub-nanomolar concentrations of probes (0.05–0.20 nm) to study receptor diffusion and aptamer binding kinetics. Examples of single EGFR trajectories on the surface of an A431 cell are shown (top image). The diffusive status of a receptor can be studied by analyzing thousands of trajectories. Additionally, aptamer binding kinetics can be assessed by analyzing the distribution of trajectory durations, which follow a single-exponential decay (black line). (**d**) Membrane receptor densities are obtained by live-cell PAINT using low nm concentrations (1–20 nm). Time-lapse sequences are recorded (on the **left**), and then a PAINT image (on the **right**) is reconstructed. Scale bar: 5 mm. (**C**) TIRF images, adapted from [75] of the plasma membrane of HeLa and BT-20 cells were recorded either in the unstimulated state, after hepatocyte growth factor (HGF) stimulation, or after activation with epidermal growth factor (EGF) (scale bar 5 m, insets 5 m, 5 m). Receptor cluster densities of MET (cyan) and EGFR (magenta). (**D**) The SIM image of s-ACE2 and h-RBD-based HeLa cells before and after incubation with dynasore, HCQ, and BafA1, adapted from [79].

Recently, more work has been published showcasing proteins requiring lipid interactions as well as other protein interactions for full catalytic or scaffolding activity [21]. Speen tyrosine kinase (Syk) is a protein found in the signaling pathways of B and myeloid cells and implicated in various cancer types, like acute myeloid leukemia [21]. Sky has been a known druggable target in the fight against hematologic malignancies, with most of the current inhibitors being ATP-competitive inhibitors with little to no long-term change for those inhibitors to offer disease-free survival. This is due to their inability to stabilize the downstream path of Syk. Ostamatinib (R788), cerdulatinib (PRT062070), TAK-659, and Entospletinib have been shown to work well in cell models but perform poorly in clinical trials due to the cells quick ability to become resistant to treatment [21]. Singaram and Sharma et al., 2023 work has shown that Syk requires interaction between its Src homology 2 (SH2) domain and a phosphatidylinositol (3,4,5) trisphosphate in the plasma membrane for full catalytic activation and scaffolding function to occur (Figure 6A) [21]. Blocking the protein–lipid interaction locks Syk in its inactive state, not allowing it to coordinate with MAP kinase and STAT, resulting in cellular disruption and cell death. Here, SMT could be used in patient samples or in vitro to test why the ATP-competitive inhibitors failed them in the way they did. Expression of Syk, FLT3, ERK1/2 and/or STAT3/5 in the presence of the inhibitors could have shed light on how the cells become resistant to treatment over time.

Epithelial lung, pancreatic, colorectal, head and neck, and medullary thyroid cancer all have a subset of patients whose cancers are driven by dysregulation of epidermal growth factor (EGF) secretion and/or overexpression/mutation of epidermal growth factor receptor (EGFR) [67]. Overexpression or mutation of EGFR drives metastasis by fueling invasion across tissue barriers, the survival of cells when not anchored, and the ability to colonize distant organs [67]. Single-molecule tracking could be used as a tool to test for the presence of overexpression of EGFR in cancers. Patient samples can be taken, and by using aptamers that are reversible, highly specific, and non-perturbing affinity probes, cell-surface receptors can be quantified and tracked to aid diagnosis. These small and reversible aptamer probes could accurately track individual EGFR motions for comparison of total EGFR density between samples [68]. In conjunction with live-cell point accumulation for imaging in nanoscale topography (PAINT), the need for persistent labeling of the EGFR is no longer needed, as are bulky or invasive probes (Figure 6B) [68]. This results in a quick experiment set up with relatively long tracking times due to the minimal photobleaching effect.

### 9.2. Drug Function and Delivery

Monitoring single molecules can help in evaluating and optimizing drug delivery systems. By labeling drug molecules or drug carriers with fluorescent tags, researchers can track their movement in vivo and study their distribution, cellular uptake, and release kinetics. This information can aid in developing more efficient drug delivery systems with improved targeting and controlled release properties. One aspect of SMT that could benefit the field of drug discovery and delivery is tracking the small molecule’s method of action, not only on the target of interest but also the off-target activity.

Aminoglycosides are a potent class of antibiotics used to treat Gram-negative and Gram-positive bacteria, which cause a whole host of various infections [69]. With their broad scope of use and potency comes an Achille’s heal of causing irreversible loss of cochlear sensory hair cells that leads to deafness in patients [70]. The mechanism of action aminoglycosides had in targeting the cochlear sensory hair cells was unknown until the use of time-lapse imaging revealed multiple proteins were responsible for the uptake of the antibiotic [70]. Tagging gentamicin with Texas Red enabled the tracking of the antibiotic in adult hearing mice. A chemo-mechanical cochleostomy revealed the antibiotic moving through the stria vascularis to the cochlea and finally being taken up by the cochlear sensory hair cells [70]. Mutagenesis of the viral antibiotic uptake was completely abolished when transmembrane channel-like protein 1 was knocked out of the cochlear cells in mice [70]. Blocking the activity of the megalin protein found in the stria vascularis by administration of an inhibitor showed a prevention of antibiotic accumulation in the cochlear ear cells. By using time-lapsed small-molecule live-cell tracking, we revealed directly how aminoglycosides attack ear cells, leading to hearing loss. Treating mice with a megalin inhibitor stopped the transport of antibiotics to the cochlear sensory hair cells, leading to a new therapeutic target for the prevention of aminoglycoside-induced deafness.

### 9.3. Biomarker Detection

Single-molecule tracking can be used to detect and analyze biomarkers, which are molecules indicative of particular diseases or conditions. By attaching fluorescent tags to biomarkers and tracking their presence and movement, researchers can develop highly sensitive diagnostic methods. This approach enables early detection and monitoring of diseases such as cancer, infectious diseases, and neurodegenerative disorders. One such biomarker that is implicated in a multitude of afflictions like depression [71] and Brugada syndrome [72] is the C-reactive protein (CRP). Part of the innate immune system, CRP, when bound to a target cell, stimulates the activity of immune cells like endothelial cells and is found to amplify proinflammatory effects caused by several mediators like endotoxins [73]. Different concentrations of CRP found in serum carry different adverse effects, with under 1 μg/mL being considered a healthy level, 1–3 μg/mL associated with cardiovascular risks, and anything over 5 μg/mL being linked to excess proinflammatory symptoms [73]. CRP has also been implicated in indirectly causing depression in individuals who also have an increase in cardiovascular disease risk [71]. A meta-analysis of individuals comparing age, HDL-cholesterol levels, BMI, and more revealed that individuals who scored higher depression levels also had higher CRP levels. Inflammation caused by CRP has been linked to many cardiac disorders, one of which is Brugada Syndrome (BrS) [72]. An inherited cardiac disorder afflicting young, healthy-looking individuals causes ventricular tachyarrhythmias, which can possibly induce syncope or sudden cardiac arrest [72]. Further exploration of this topic led to further evidence supporting the hypothesis that CRP has the ability to activate blood monocytes and vessel-wall endothelial cells [74]. Currently, CRP is the most useful inflammatory biomarker in the prediction of cardiovascular events, and studying CRP expression, release, and method of action via SMT can open the doors to new possibilities for mitigating cardio-vascular stress through inflammation. If the different levels of CRP in the serum cause different afflictions, taking patient samples of monocytes, cells that release endotoxins, and vessel-wall endothelial cells and monitoring under which conditions those cells can lead to and quantifying the production of CRP could directly link to the root cause of cardiovascular disease, depression, and other afflictions.

### 9.4. Unraveling Disease Mechanisms

Single-molecule tracking can contribute to our understanding of various disease mechanisms. By tracking the behavior of specific molecules associated with diseases, researchers can identify aberrant cellular processes, dysfunctional molecular interactions, and altered transport mechanisms. This information can provide insights into disease progression, identify potential therapeutic targets, and aid in the development of novel treatment strategies.

Receptor tyrosine kinases (RTKs) are transmembrane receptors that are first in line to receive signals for cellular communication, cellular growth, and motility [75]. When RTKs bind to their respective ligands, they dimerize with one another, which leads to autophosphorylation of their inner membrane motif, which initiates a downstream signaling cascade [75]. Overexpression or mutation of RTKs leads to the start and progression of a multitude of diseases, one of which is cancer. With a high incidence and implication rate, RTKs have been the focus of many pharmaceutical therapies, and SMT, could lead to further understanding how RTKs are implemented in these various diseases. Disfunctions in HER2, a member of the RTK family, were found to cause inflammation and cancer [76]. With no known ligand to activate HER2, it has been classified as an orphan receptor, relying upon dimerization of itself with another receptor for activation [76]. Stimulation of Hela cells with epithelial growth factor (EGF) or transforming growth factor alfa (TGFα) leads to the homodimerization of EGFR and HER2 [76]. This process was tracked using a Cy3B-labeled anti-HER2 nanobody that showed a resulting decrease in total diffusion coefficients upon ligand addition to the system [76]. As the total diffusion coefficient decreased, the number of immobilized receptors increased, indicating that HER2 was immobilized by dimerization [76]. This was cross-referenced with Western blot analysis, which confirmed the increase in HER2 phosphorylation level over multiple time points (up to 25 min) when ligand was present. RTK not only plays a role in cancer development but also has profound effects on the outcomes of patients whose cancer progresses through mutations and changes in RTK expression or activation. When patients become resistant to their current treatment, the resistance typically comes from the RTKs ability to circumvent the single blocked pathway by upregulating a separate pathway, which leads to the same result or mutation of the inhibited protein [75]. This is due to our cells having many redundant signaling pathways, which under normal conditions means no harm, but in cancer, blocking a single pathway makes it much more difficult to treat. SMT and super-resolution microscopy were used to monitor hepatocyte growth factor receptor (MET) and EGFR activation in Hela and BT-20 cells (Figure 6C) [75]. It is believed that MET overexpression leads to EGFR-TKI resistance in cancer patients, and more needs to be understood about the relationship between MET and EGFR. SMT provided evidence that MET and EGFR heteromeric clusters form in the absence and presence of their respected ligands. This clustered formation was also related to the expression levels of the receptors.

Hepatocellular carcinoma (HCC), a malignant tumor that can be found on the liver, is the fifth leading cause of tumor death, with an incidence rate of over a million patients a year [77]. The tumor can grow well on the liver due to its ability to divert blood from one of the two main arteries. This has led to a promising treatment: transcatheter arterial chemoembolization (TACE), which induces hypoxia in the tumor, necrosis, and tumor shrinkage [77]. Although this procedure was promising, its long-term efficacy is not satisfactory, with a five-year survival rate of less than 10%. The stress of hypoxia on the tumor results in the tumor’s ability to undergo a series of metabolic changes that enable the tumor to induce the formation of new blood vessels to survive (neovascularization). MTA1, a component of the nucleosome remodeling and histone deacetylation (NuRD) complex, was shown to be overexpressed in multiple cancer types: gastric, breast, colorectal, and others [77]. This increase in protein expression has been linked to increased cell motility, growth, and metastasis, making MTA1 a potential indicator of the aggressiveness of a particular tumor. One role that this protein might be implicated in is angiogenesis. HIF-1 (hypoxia-inducible factor-1) is a transcription factor that mediates the cellular response to hypoxia. MTA1s’ relationship to HIF-1 was unknown [77]. It was found that the expression of HIF-1 was enhanced in the presence of MTA1 only under hypoxic conditions. The co-immunoprecipitation assay showed direct interaction with the HIF-1α subunit and MTA1 [77]. This evidence indicated the role of MTA1 as an active angiogenic regulator through the direct binding of HIF-1, which led to its proangiogenic capability.

HER2 receptor, SMT could help determine how the activity of HER2 on the surface of the cells obtained from patients plays a role in cancer progression. This could help determine if HER2-positive cancer treatment will be beneficial for the patient and not cause any adverse effects like further mutations or resistance. SMT could also aid in determining the resistance pathway a specific patient’s cancer has gained. Visualizing RTKs in patient samples, like EGFR and MET, and how they interact with each other in the presence of an inhibitor or ligand, could lead to other forms of more effective treatment. A big role that SMT could play is in determining how tumor cells overcome treatment and continue to grow. Taking HCC as an example, HIF-1 was known to mediate the cells ability to find and induce the growth of new blood vessels to feed themselves [77]. SMT in this case could show what MTA1 interacts with, leading to new possibilities for PPI inhibitors or changes in treatment approaches.

### 9.5. Virus and Pathogen Studies

Single-molecule tracking techniques can provide valuable insights into the behavior of viruses and other pathogens. By labeling viral particles or pathogen components, researchers can track their interactions with host cells, study viral entry mechanisms, and analyze the efficacy of antiviral treatments. This information can aid in the development of diagnostic tools, vaccines, and antiviral therapies.

Much stress has been placed on the scientific community to resolve every aspect of the virus’s behavior in the wake of the COVID-19 pandemic. A large portion of time is spent understanding how viruses are endocytosed into our cells. Structure illumination microscopy (SIM) imaging has resolved the interaction between a receptor binding domain (RBD) and angiotensin-converting enzyme 2 (ACE2) with rhodamine/cyanine dyes [78]. SIM imaging tracked, in real time, the endocytosis pathway taken when RBD and ACE2 interact, showing recognition of the two proteins, internalization of the RBD-ACE2 complex, RAB GTPases bearing vesical formation, transport, degradation, and finally downregulation of ACE2 [78]. RBD from wild-type SARS-CoV-2 spike protein and an ACE2 protein were labeled with organic dyes, and the entire process of endocytosis was tracked like previously by multi-color SIM super-resolution imaging [78]. Using the RBD from the SARS-CoV-2 spike protein induced a different initial step in endocytosis, where this RBD was able to initiate endocytosis almost synergistically with ACE2 [78]. Once the RBD was added to the system, within 20 min it was taken up as cargo; this internalization timeline was much shorter than without the RBD present. Multi-color SIM super-resolution imaging tracked the movements and colocalization of the internalized spike protein and ACE2 from fusion of vesicles, vesical movement along microtubules, and cargo exchange between early endosomes and late endosomes (Figure 6D) [78]. These results have demonstrated that viruses are internalized and degraded by the lysosome; however, the DNA or RNA from the virus drives the creation of new viruses to be released from the lysosome.

As we uncover more in-depth information on how cells internalize viruses, the other half of the equation needs to be explored as well. What makes viruses so efficient at fusion and internalization into the cell? Electron microscopy (EM), super-resolution microscopy, and SPT were utilized to examine a large collection of individual vaccinia virus (VACV) particles in the hope of understanding virus binding, fusion orientation, and the distribution of binding and fusion proteins to link fusion-protein distribution and fusion activity [79]. Scanning electron microscopy (SEM) and transmission electron microscopy (TEM) both revealed that VACV mature virions bind to the plasma membrane exclusively from the side of the virion, while dropping the pH of the system forced the fusion of the virion to occur almost exclusively at the tip of the mature virion [79]. These data were consistent with previously published reports, leading to the indication that viral membrane proteins could be organized into functional domains. Proteins found in the virion were tagged with fluorescent proteins to identify virion orientation (core protein A4 was tagged with mCherry) and its relation to a membrane marker (protein A1319 was tagged with eGFP) [79]. SIM and single-particle averaging were used to generate models of the distribution of the aforementioned proteins during binding, entry, and fusion of the virions [79]. These models depict “hot zones” where the binding proteins reside only at the sides of the virions and the entry fusion complexes are all clustered at the top and bottom of the virions, independent of orientation. Mutations and deletions of individual binding and fusion proteins showed these sequestered complexes were actually organized in distinct functional domains around the viral membrane and that the polarized distribution of the entry fusion complexes relied on all the proteins being intact or present. Utilizing single-particle analysis in combination with other microscopy tools has opened the door to understanding how protein complexes found on the virion’s plasma membrane correlate with virus function. This deepens our understanding of how and why viruses are so efficient in their ability to infect.

## 10. Making SMT a More Robust Technique

Single-molecule tracking has come a long way in recent years, affording better fluorophores, labeling techniques, continuous revisions to analysis for better determination of protein behaviors, and much more. From the first step to the last step, the processes of tracking a single molecule have continuously evolved to make SMT a more robust and user-friendly technique.

The process of labeling proteins with fluorescent probes or tags has been a point of continuous evolution. These labels come in a wide variety of colors and can be expressed with the POI or attached after the protein is expressed through techniques such as immunofluorescence or genetic fusion. With the introduction of self-labeling enzymes like HaloTags to the world of fluorescence imaging, more precise tracking of POIs can be achieved by diving deeper into molecular processes like subcellular protein translocation, PPIs, and much more [80]. With the Halo Tag’s success, other authors sought to make it a tool that could be used in broader applications. Where the initial Halo Tag was essentially irreversible [80], modifying the haloalkane dehalogenase (HaloTag) back to an enzyme that could reversibly bind to the synthetic ligands left a protein that allowed for a continuous exchange of photobleached chromophores, which in turn enabled extended time-lapsed imaging [81]. reHaloTag was created to overcome the photobleaching seen when HaloTags were used for long-term time-lapse super-resolution imaging [81]. Improvements in labeling techniques did not stop there. The delivery of dyes, probes, and even labeled proteins into cells has been investigated for improving multi-channel/color SMT. Current approaches include microinjection of purified and labeled proteins into cells but require very specialized equipment, resulting in a labor-intensive process [41,82]. Cuvette electroporation (CEP), on the other hand, has shown promising results in delivering dye-labeled molecules into a cell for SMT [83,84], but does show limitations when looking to deliver multiple probes at once. To overcome this limitation, Chen et al., 2021 have developed a nanopore-electroporation (NanoEP) delivery technique that allowed for the delivery of multiple organic dye-labeled proteins for multicolor SMT. Their method induced the formation of tiny pores in the cell membrane that help maintain the structure of the cell membrane [85]. The biggest factor that makes NanoEP much more effective than traditional CEP is the addition of electrophoresis as the main transport mechanism of the charged cargo, which affords a 10-to-100-fold increase in cargo delivery efficiency [85]. This combination gave them the possibility to concurrently visualize the EGFR signaling transduction pathway by NanoEP delivery of GrB2 and RBD, while SOS and Src were transiently expressed by plasmid transfection [85].

These advancements in protein labeling and the delivery of dyes or protein dye conjugates all serve to make live-cell imaging a much more robust and useful technique for determining protein behaviors. SMT not only needs labeled proteins to follow and detect but also requires a powerful microscope to detect them. Commonly, SMT techniques, such as TIRF microscopy or super-resolution microscopy, can be employed to achieve high spatial resolution and single-molecule sensitivity; however, scientists and researchers seek to improve on the current method.

As mentioned previously, photobleaching of proteins and fluorophores is a very common issue when proteins of interest are exposed for an extended period of time. This is especially true in applications like SMT, where the goal is to track the movement of a target over a time course. Others have tried to overcome this photobleaching effect by mutation; however, a simpler alternative can be had. By adding low concentrations of dissolved oxygen to a reducing-plus-oxidizing system, the photobleaching/photo blinking of the fluorophore can almost be completely negated [86]. This allows for proteins of interest to be tracked for upwards of 12,000 frames or a 7 min video, depending on the rate of capture [86]. Since this whole system requires such a small amount of dissolved oxygen, reducer, and oxidizer, it has minimal detrimental effects on the living cell. In some cases, proteins do not function on an individual protein level but form protein complexes that are necessary for full protein activity or stability. In many cases, this has been difficult to identify through microcopy, so in Basu et al., 2018, the authors wanted to create a system where this identification of the formation of a protein complex was possible. By utilizing FRET pairs under TIRFM, we have the ability to visualize in real time a protein complex forming and track the complex [87]. In this specific case, the FRET pairs were tuned to enhance the photostability of the individual fluorophores, allowing the FRET pairs to compete with the traditional photobleaching kinetic pathway plaguing many. As proteins become better labeled, the apparatuses detecting and tracking these proteins have also continuously evolved. Much effort has been made to bring down the already tight resolution of 100 nm to 200 nm that TIRF carries. One such effort came in the form of combining TIRFM with super-resolution fluorescence microscopy, specifically structured illumination microscopy (SIM). The combination of an ultrahigh numerical aperture (NA) lens and TIRFM gave a very low resolution of 84 nm resolution [18]. This low resolution allowed for the tracking of cortical filamentous actin associations with either myosin IIA, paxillin, or clathrin [18]. The high spatial and temporal resolution gives us the ability to confidently measure structures like clathrin-coated pits throughout their life cycle. The use of nonlinear SIM (using pattered activation (PA NL-SIM)) gave even greater spatial and temporal resolution down to 45–62 nm, which allowed for live tracking of actin cytoskeleton remodeling and other protein dynamics at the cell surface. The combination of PA NL-SIM and lattice light sheet microscopy methods allowed for the 3D tracking of single proteins across the entire volume of a cell. Diffusion, dynamics in the cytoskeleton, movement of mitochondria, and trafficking of vesical to and from the Golgi were all tracked with a relative five-fold higher resolution than from widefield microscopy. This method has been further enhanced with the addition of a switchable spatial light modulator [88]. This addition permitted the quantitative evaluation of single tracer molecules and native messenger ribonucleoprotein particles (mRNPs) in salivary gland cell nuclei with the cells suspended in solution. Many researchers have started to use other microscopy techniques other than TIRFM. An interesting approach to SMT and tracking PPIs comes in the form of a combination of a protein-fragment complementation assay and photoactivated localization microscopy (PALM) [89]. The protein-fragment complementation assay works based on fluorescent complementation reporters (each protein has a fully formed fluorophore instead of half of a fluorophore that needs to associate with its complementary half to give off fluorescence). Among the different protein-fragment complementation assays, bimolecular fluorescence complementation (BiFC) was used in combination with PALM to image and track the subcellular distribution of individual interactions between the two POIs with high special and temporal resolution. Much discussion has been had on how to track single partials on multiple axes [90,91,92,93]. One new method being implemented across the field is real-time 3D single particle tracking (RT-3D-SPT) [32]. This method has the hope of untethering single molecules from one plane by using active feedback to lock onto a free protein or particle and track its movements across multiple z-axis planes. It has been shown to track a particle continuously with up to a photon-limited temporal resolution.

Since the late 1980s, algorithms have been written and published to track single protein movements with high precision [94]. Having a base to work with, multiple scientists have utilized, modified, and built upon Gellas et al., 1988’s work to further their own research [95]. We have come a long way since the 1980s, leaving no stone unturned when it comes to improving colocalization and quantitative analysis for more accurate data acquisition and broadening the applicational range of SMT.

One selected author believes that no matter how good the experiment can be, the major limiting factors for data acquisition and analysis are the current imaging processing and/or particle tracking algorithms [96]. Kou et al., 2018 explain that their method of building a graphing-processing unit based on a compute unified device architecture in conjunction with a home-build centrifugal force microscope allows them to obtain 1 nm resolution in 3D. Some chose to focus on upgrading hardware for better temporal resolution. Small et. al., 2016 used mathematics to determine the exact desired number of expressed fluorophores to have a high enough density without sacrificing precision due to overlap in the signal. The ability to track either single or multi-fluorophore proteins with high precision came down to having the minimum fluorophore space be relative to the point spread function (PSF) of the individual POI so that the overlap in the PSF does not shift the center of the POI by more than the localization distance [97]. Updates to current software and the introduction of new software to evaluate particle trajectories better than the previous versions are a never-ending challenge. There are a few limitations with the current model-fitting programs (based on the particle mean square displacement) for the evaluation of particle trajectories for SMT, one of which is that the model fitting is too simple. To properly evaluate the motion of a single particle, it is necessary to measure the instantaneous values of motion over time at every individual frame [98]. This allows for not just determining one path for the particle but also generating multiple paths for a single particle and the likelihood of each path [98]. The Biggles tracker was developed as an automatic Bayesian inference-based, Gibbs-sampler, and global estimate of particle trajectories that globally optimized a spatiotemporal solution for real-world tracking. Since it has the ability to generate multiple trajectories and probabilities for each trajectory, it can estimate the uncertainty of the tracked solution and provide alternative solutions. This translates to quantifying errors in biophysical quantities like dwell time and diffusion coefficients. Hirsch et al., 2019 are not alone in making improvements to the global-based fitting algorithm, with Li et. al., 2022, publishing globLoc. A graphics processing unit based on the global fitting algorithm is used to improve the precision of 3D localization ratiometric multicolor imaging [99].

As the process of SMT becomes more robust, there is hope to use this method as a large-scale automation for single-molecule screening [100].

## 11. Limitations and Outlook

TIRF microscopy is a powerful imaging technique that allows researchers to study events occurring at or near the surface of a specimen with high spatial resolution [4,13,35,93]. Although TIRFM is one of the most common methods used when tracking single molecules, TIRFM has some limitations. First of which is its limited penetration depth. TIRF microscopy has been primarily designed to visualize events that occur at or near the interface between a specimen and a glass coverslip. The penetration depth of the evanescent wave is typically around 100–200 nanometers [18,20,35,101] which means that it cannot visualize structures deeper within the specimen. This limits TIRFM’s usability to very thin tissue samples or cells. TIRFM is less suitable for studying intracellular events or structures that are located deeper within tissue samples. This could become a limiting factor for using TIRFM as a diagnostic tool. Although TIRF microscopy provides excellent spatial resolution [30,46,102], the drawback of having a low penetration depth comes from the restricted field of view. Since only a thin region adjacent to the coverslip Is illuminated by the evanescent wave, studying large-scale cellular processes, or trying to capture a broader context of cellular events is almost impossible. Due to the restricted field of view, controlling the imaging depth precisely can be challenging. The depth of the evanescent wave is influenced by various factors, including the refractive index of the specimen and the angle of incidence. Achieving precise control over the imaging depth can be difficult, and variations in these factors can affect the accuracy of quantitative measurements. TIRF microscopy, which utilizes powerful lasers to excite fluorescent proteins and dyes in combination with a thin illumination area, results in a high tendency for the laser to photobleach the fluorophore and/or become phototoxic to the cell [22,63,102,103]. Continuous illumination at high-intensity levels can lead to photobleaching, where the fluorophores lose their ability to fluoresce. Additionally, the high-intensity illumination used in TIRF microscopy can also cause phototoxicity, leading to cellular damage and affecting the viability of live specimens. Despite these limitations, TIRF microscopy remains a valuable tool for studying surface-associated phenomena with high spatial resolution, and researchers continue to explore ways to overcome these challenges and expand its applications. As mentioned above, new microscopy, quantification, and labeling techniques are being implemented to overcome the limitations of TIRFM, like STORM, PAINT, and SIM, to name a few.

The future of signal-molecule tracking holds several exciting possibilities for further advancements and expanded applications. Efforts are being made to enhance the penetration depth of TIRF microscopy to enable imaging deeper within specimens. Researchers are exploring novel techniques, such as structured illumination and adaptive optics, to extend the imaging range and overcome the current limitations. TIRF microscopy has primarily been used for single-color imaging. However, advancements in fluorophore development and labeling techniques are enabling the expansion of TIRF microscopy to multicolor imaging. This will allow researchers to simultaneously visualize multiple cellular components or dynamic processes with different fluorophores [81,87,98,104]. Super-resolution microscopy techniques, such as STED [14] and stochastic optical reconstruction microscopy (STORM) [45,76,89] have revolutionized the field of cellular imaging by achieving resolutions beyond the diffraction limit of TIRFM. Integrating super-resolution capabilities with TIRF microscopy would enable researchers to study surface-associated structures and events with unprecedented detail. Live-cell imaging using TIRF microscopy has immense potential for studying dynamic processes at the cell membrane in real time. Advancements in imaging techniques, such as faster cameras, improved fluorophores with higher photostability, and better temperature and environmental control systems, will further enhance the capabilities of live-cell TIRF microscopy [86,96,98,99]. Combining TIRF microscopy with other imaging modalities, such as FRET [4,87], STORM [89], or single-molecule imaging, can provide complementary information about cellular processes and interactions. Integrating TIRF microscopy with techniques like atomic force microscopy (AFM) [105] or electron microscopy (EM) [79] can enable simultaneous visualization of cellular structures at different resolutions. TIRF microscopy has significant potential in drug discovery and diagnostics, particularly for studying membrane receptor dynamics, protein–protein interactions, and membrane trafficking. By providing detailed insights into these processes, TIRF microscopy can aid in the development of targeted therapeutics and diagnostic tools. TIRF microscopy can be combined with microfluidics and lab-on-a-chip systems to create powerful platforms for high-throughput screening, cell sorting, and single-cell analysis [106,107,108,109]. This integration allows for precise control over the cellular microenvironment and enables the study of dynamic processes in a more physiologically relevant context. These future perspectives of TIRF microscopy highlight the ongoing advancements and potential applications that will further enhance our understanding of cellular processes and contribute to various fields, including cell biology, biophysics, pharmacology, and biomedical research.

## 12. Conclusions

Single-molecule tracking in live cells allows researchers to gain insights into fundamental biological processes such as protein–protein interactions, membrane dynamics, receptor trafficking, and cytoskeletal dynamics. It has provided valuable information about the heterogeneity and complexity of cellular processes, revealing behaviors that would not be apparent in ensemble measurements. Single-molecule tracking has come a long way since the technique first came into use in the 1980s and is now tracking single molecules for extended periods of time in a live cell. Tracking multiple proteins, lipids, and organelles at the same time with incredible spatial-temporal resolution. It has been used to understand why some drugs have adverse side effects or why cells become resistant to inhibitors. Single-molecule tracking has even resolved the different ways and mechanisms by which viruses attack our own cells. TIRM, in combination with EM, PALM, STORM, and other microscopy techniques, makes it a powerful tool for uncovering the most important aspect of a cell.

We believe that single-molecule tracking has much more to offer than just being a tool used to study cultured cells. With a few modifications to the current assembly, comb-microscopy techniques, and sorting out ways to make it a more accessible tool to be used in research hospitals, it can make medical diagnoses and determining the efficacy of treatment that much more accurate. This technique has significantly contributed to our understanding of cell biology and has the potential to uncover new insights into the functioning and fundamentals of living systems.

## Data Availability

This review is based on publicly accessible literature and databases.

## References

[1] Kuhn T., Hettich J., Davtyan R., Gebhardt J.C.M. (2021). Single Molecule Tracking and Analysis Framework Including Theory-Predicted Parameter Settings. Sci. Rep..

[2] Hou S., Exell J., Welsher K. (2020). Real-Time 3D Single Molecule Tracking. Nat. Commun..

[3] Bayle V., Fiche J.-B., Burny C., Platre M.P., Nollmann M., Martinière A., Jaillais Y. (2021). Single-Particle Tracking Photoactivated Localization Microscopy of Membrane Proteins in Living Plant Tissues. Nat. Protoc..

[4] Kusumi A., Tsunoyama T.A., Hirosawa K.M., Kasai R.S., Fujiwara T.K. (2014). Tracking Single Molecules at Work in Living Cells. Nat. Chem. Biol..

[5] Yu Y., Li M., Yu Y. (2019). Tracking Single Molecules in Biomembranes: Is Seeing Always Believing?. ACS Nano.

[6] Zhang M.-L., Ti H.-Y., Wang P.-Y., Li H. (2021). Intracellular Transport Dynamics Revealed by Single-Particle Tracking. Biophys. Rep..

[7] Li N., Zhao R., Sun Y., Ye Z., He K., Fang X. (2017). Single-Molecule Imaging and Tracking of Molecular Dynamics in Living Cells. Natl. Sci. Rev..

[8] Milenkovic L., Weiss L.E., Yoon J., Roth T.L., Su Y.S., Sahl S.J., Scott M.P., Moerner W.E. (2015). Single-Molecule Imaging of Hedgehog Pathway Protein Smoothened in Primary Cilia Reveals Binding Events Regulated by Patched1. Proc. Natl. Acad. Sci. USA.

[9] Thrall E.S., Kath J.E., Chang S., Loparo J.J. (2017). Single-Molecule Imaging Reveals Multiple Pathways for the Recruitment of Translesion Polymerases after DNA Damage. Nat. Commun..

[10] Yu D., Baird M.A., Allen J.R., Howe E.S., Klassen M.P., Reade A., Makhijani K., Song Y., Liu S., Murthy Z. (2015). A Naturally Monomeric Infrared Fluorescent Protein for Protein Labeling In Vivo. Nat. Methods.

[11] Stehr F., Stein J., Bauer J., Niederauer C., Jungmann R., Ganzinger K., Schwille P. (2021). Tracking Single Particles for Hours via Continuous DNA-Mediated Fluorophore Exchange. Nat. Commun..

[12] Hellweg L., Edenhofer A., Barck L., Huppertz M.-C., Frei M.S., Tarnawski M., Bergner A., Koch B., Johnsson K., Hiblot J. (2023). A General Method for the Development of Multicolor Biosensors with Large Dynamic Ranges. Nat. Chem. Biol..

[13] Jensen E.C. (2013). Overview of Live-Cell Imaging: Requirements and Methods Used. Anat. Rec. Adv. Integr. Anat. Evol. Biol..

[14] Prakash K., Diederich B., Heintzmann R., Schermelleh L. (2022). Super-Resolution Microscopy: A Brief History and New Avenues. Philos. Trans. R. Soc. Math. Phys. Eng. Sci..

[15] Weiss S. (2000). Shattering the Diffraction Limit of Light: A Revolution in Fluorescence Microscopy?. Proc. Natl. Acad. Sci. USA.

[16] Lee J., Miyanaga Y., Ueda M., Hohng S. (2012). Video-Rate Confocal Microscopy for Single-Molecule Imaging in Live Cells and Superresolution Fluorescence Imaging. Biophys. J..

[17] Segers-Nolten G.M.J., Wyman C., Wijgers N., Vermeulen W., Lenferink A.T.M., Hoeijmakers J.H.J., Greve J., Otto C. (2002). Scanning Confocal Fluorescence Microscopy for Single Molecule Analysis of Nucleotide Excision Repair Complexes. Nucleic Acids Res..

[18] Li D., Shao L., Chen B.-C., Zhang X., Zhang M., Moses B., Milkie D.E., Beach J.R., Hammer J.A., Pasham M. (2015). Extended-Resolution Structured Illumination Imaging of Endocytic and Cytoskeletal Dynamics. Science.

[19] Sönnichsen C., Geier S., Hecker N.E., Von Plessen G., Feldmann J., Ditlbacher H., Lamprecht B., Krenn J.R., Aussenegg F.R., Chan V.Z.-H. (2000). Spectroscopy of Single Metallic Nanoparticles Using Total Internal Reflection Microscopy. Appl. Phys. Lett..

[20] Liu Z., Lavis L.D., Betzig E. (2015). Imaging Live-Cell Dynamics and Structure at the Single-Molecule Level. Mol. Cell.

[21] Singaram I., Sharma A., Pant S., Lihan M., Park M.-J., Pergande M., Buwaneka P., Hu Y., Mahmud N., Kim Y.-M. (2023). Targeting Lipid–Protein Interaction to Treat Syk-Mediated Acute Myeloid Leukemia. Nat. Chem. Biol..

[22] Chen Q., Fang H., Shao X., Tian Z., Geng S., Zhang Y., Fan H., Xiang P., Zhang J., Tian X. (2020). A Dual-Labeling Probe to Track Functional Mitochondria–Lysosome Interactions in Live Cells. Nat. Commun..

[23] Hong J., Li Q., Xia Q., Feng G. (2021). Real-Time and High-Fidelity Tracking of Lysosomal Dynamics with a Dicyanoisophorone-Based Fluorescent Probe. Anal. Chem..

[24] Sen B., Peng S., Woods D.M., Wistuba I., Bell D., El-Naggar A.K., Lai S.Y., Johnson F.M. (2012). STAT5A-Mediated SOCS2 Expression Regulates Jak2 and STAT3 Activity Following c-Src Inhibition in Head and Neck Squamous Carcinoma. Clin. Cancer Res..

[25] Van Zijl F., Krupitza G., Mikulits W. (2011). Initial Steps of Metastasis: Cell Invasion and Endothelial Transmigration. Mutat. Res. Mutat. Res..

[26] Kato G.J., Piel F.B., Reid C.D., Gaston M.H., Ohene-Frempong K., Krishnamurti L., Smith W.R., Panepinto J.A., Weatherall D.J., Costa F.F. (2018). Sickle Cell Disease. Nat. Rev. Dis. Prim..

[27] Meyer J., Priemel M., Rolvien T., Frosch K.-H., Schlickewei C., Yarar-Schlickewei S. (2023). The Diagnostic Challenge of Osteoid Osteoma in the Bones of the Hand—A Case Series. Diagnostics.

[28] Pongsuvareeyakul T., Saipattranusorn K., Sukpan K., Suprasert P., Khunamornpong S. (2023). Clear-Cell Mesothelioma of Uterine Corpus: Diagnostic Challenges in Intraoperative Frozen Sections. Diagnostics.

[29] Huang T.-Y., Feng P.-C., Wang Y.-C., Su C.-Y. (2022). Differential Diagnosis of Thoracoacromial Artery Pseudoaneurysm from Shoulder Inflammatory Pseudotumor: A Case Report. Diagnostics.

[30] Ma Y., Wang X., Liu H., Wei L., Xiao L. (2019). Recent Advances in Optical Microscopic Methods for Single-Particle Tracking in Biological Samples. Anal. Bioanal. Chem..

[31] Ye Z., Wang X., Xiao L. (2019). Single-Particle Tracking with Scattering-Based Optical Microscopy. Anal. Chem..

[32] Hou S., Johnson C., Welsher K. (2019). Real-Time 3D Single Particle Tracking: Towards Active Feedback Single Molecule Spectroscopy in Live Cells. Molecules.

[33] Yang T.T., Tran M.N.T., Chong W.M., Huang C.-E., Liao J.-C. (2019). Single-Particle Tracking Localization Microscopy Reveals Nonaxonemal Dynamics of Intraflagellar Transport Proteins at the Base of Mammalian Primary Cilia. Mol. Biol. Cell.

[34] Cui Y., Yu M., Yao X., Xing J., Lin J., Li X. (2018). Single-Particle Tracking for the Quantification of Membrane Protein Dynamics in Living Plant Cells. Mol. Plant.

[35] Kudalkar E.M., Davis T.N., Asbury C.L. (2016). Single-Molecule Total Internal Reflection Fluorescence Microscopy. Cold Spring Harb. Protoc..

[36] Iino R., Koyama I., Kusumi A. (2001). Single Molecule Imaging of Green Fluorescent Proteins in Living Cells: E-Cadherin Forms Oligomers on the Free Cell Surface. Biophys. J..

[37] Sako Y., Minoghchi S., Yanagida T. (2000). Single-Molecule Imaging of EGFR Signalling on the Surface of Living Cells. Nat. Cell Biol..

[38] Chen J., Zhang Z., Li L., Chen B.-C., Revyakin A., Hajj B., Legant W., Dahan M., Lionnet T., Betzig E. (2014). Single-Molecule Dynamics of Enhanceosome Assembly in Embryonic Stem Cells. Cell.

[39] Ritter J.G., Veith R., Veenendaal A., Siebrasse J.P., Kubitscheck U. (2010). Light Sheet Microscopy for Single Molecule Tracking in Living Tissue. PLoS ONE.

[40] Hiramoto-Yamaki N., Tanaka K.A.K., Suzuki K.G.N., Hirosawa K.M., Miyahara M.S.H., Kalay Z., Tanaka K., Kasai R.S., Kusumi A., Fujiwara T.K. (2014). Ultrafast Diffusion of a Fluorescent Cholesterol Analog in Compartmentalized Plasma Membranes. Traffic.

[41] Buwaneka P., Ralko A., Liu S.-L., Cho W. (2021). Evaluation of the Available Cholesterol Concentration in the Inner Leaflet of the Plasma Membrane of Mammalian Cells. J. Lipid Res..

[42] Termini C.M., Gillette J.M. (2017). Tetraspanins Function as Regulators of Cellular Signaling. Front. Cell Dev. Biol..

[43] Espenel C., Margeat E., Dosset P., Arduise C., Le Grimellec C., Royer C.A., Boucheix C., Rubinstein E., Milhiet P.-E. (2008). Single-Molecule Analysis of CD9 Dynamics and Partitioning Reveals Multiple Modes of Interaction in the Tetraspanin Web. J. Cell Biol..

[44] Kaksonen M., Roux A. (2018). Mechanisms of Clathrin-Mediated Endocytosis. Nat. Rev. Mol. Cell Biol..

[45] Scott B.L., Sochacki K.A., Low-Nam S.T., Bailey E.M., Luu Q., Hor A., Dickey A.M., Smith S., Kerkvliet J.G., Taraska J.W. (2018). Membrane Bending Occurs at All Stages of Clathrin-Coat Assembly and Defines Endocytic Dynamics. Nat. Commun..

[46] Anantharam A., Onoa B., Edwards R.H., Holz R.W., Axelrod D. (2010). Localized Topological Changes of the Plasma Membrane upon Exocytosis Visualized by Polarized TIRFM. J. Cell Biol..

[47] Wang H., Vilela M., Winkler A., Tarnawski M., Schlichting I., Yumerefendi H., Kuhlman B., Liu R., Danuser G., Hahn K.M. (2016). LOVTRAP: An Optogenetic System for Photoinduced Protein Dissociation. Nat. Methods.

[48] Li K., Rittase W., Yuan Z., Zhu C. (2019). Single-Molecule Investigations of T-Cell Activation. Curr. Opin. Biomed. Eng..

[49] Rossboth B., Arnold A.M., Ta H., Platzer R., Kellner F., Huppa J.B., Brameshuber M., Baumgart F., Schütz G.J. (2018). TCRs Are Randomly Distributed on the Plasma Membrane of Resting Antigen-Experienced T Cells. Nat. Immunol..

[50] Lee J.Y., Finkelstein I.J., Arciszewska L.K., Sherratt D.J., Greene E.C. (2014). Single-Molecule Imaging of FtsK Translocation Reveals Mechanistic Features of Protein-Protein Collisions on DNA. Mol. Cell.

[51] Jean N.L., Rutherford T.J., Löwe J. (2020). FtsK in Motion Reveals Its Mechanism for Double-Stranded DNA Translocation. Proc. Natl. Acad. Sci. USA.

[52] Taguchi H., Ueno T., Tadakuma H., Yoshida M., Funatsu T. (2001). Single-Molecule Observation of Protein–Protein Interactions in the Chaperonin System. Nat. Biotechnol..

[53] Verweij F.J., Revenu C., Arras G., Dingli F., Loew D., Pegtel D.M., Follain G., Allio G., Goetz J.G., Zimmermann P. (2019). Live Tracking of Inter-Organ Communication by Endogenous Exosomes In Vivo. Dev. Cell.

[54] Zhang M.-Q., Wang Z.-G., Fu D.-D., Zhang J.-M., Liu H.-Y., Liu S.-L., Pang D.-W. (2022). Quantum Dots Tracking Endocytosis and Transport of Proteins Displayed by Mammalian Cells. Anal. Chem..

[55] Yang L.-Z., Wang Y., Li S.-Q., Yao R.-W., Luan P.-F., Wu H., Carmichael G.G., Chen L.-L. (2019). Dynamic Imaging of RNA in Living Cells by CRISPR-Cas13 Systems. Mol. Cell.

[56] Wang H., Nakamura M., Abbott T.R., Zhao D., Luo K., Yu C., Nguyen C.M., Lo A., Daley T.P., La Russa M. (2019). CRISPR-Mediated Live Imaging of Genome Editing and Transcription. Science.

[57] Andrieux P., Chevillard C., Cunha-Neto E., Nunes J.P.S. (2021). Mitochondria as a Cellular Hub in Infection and Inflammation. Int. J. Mol. Sci..

[58] Kramer P., Bressan P. (2018). Our (Mother’s) Mitochondria and Our Mind. Perspect. Psychol. Sci..

[59] Qin J., Guo Y., Xue B., Shi P., Chen Y., Su Q.P., Hao H., Zhao S., Wu C., Yu L. (2020). ER-Mitochondria Contacts Promote MtDNA Nucleoids Active Transportation via Mitochondrial Dynamic Tubulation. Nat. Commun..

[60] Wiseman R.L., Mesgarzadeh J.S., Hendershot L.M. (2022). Reshaping Endoplasmic Reticulum Quality Control through the Unfolded Protein Response. Mol. Cell.

[61] Schwarz D.S., Blower M.D. (2016). The Endoplasmic Reticulum: Structure, Function and Response to Cellular Signaling. Cell. Mol. Life Sci..

[62] Oakes S.A., Papa F.R. (2015). The Role of Endoplasmic Reticulum Stress in Human Pathology. Annu. Rev. Pathol. Mech. Dis..

[63] Eördögh Á., Paganini C., Pinotsi D., Arosio P., Rivera-Fuentes P. (2020). A Molecular Logic Gate Enables Single-Molecule Imaging and Tracking of Lipids in Intracellular Domains. ACS Chem. Biol..

[64] Wenzel E.M., Elfmark L.A., Stenmark H., Raiborg C. (2022). ER as Master Regulator of Membrane Trafficking and Organelle Function. J. Cell Biol..

[65] Wang X., Wang H., Xu B., Huang D., Nie C., Pu L., Zajac G.J.M., Yan H., Zhao J., Shi F. (2021). Receptor-Mediated ER Export of Lipoproteins Controls Lipid Homeostasis in Mice and Humans. Cell Metab..

[66] Wahab Khattak F., Salamah Alhwaiti Y., Ali A., Faisal M., Siddiqi M.H. (2021). Protein-Protein Interaction Analysis through Network Topology (Oral Cancer). J. Healthc. Eng..

[67] Uribe M.L., Marrocco I., Yarden Y. (2021). EGFR in Cancer: Signaling Mechanisms, Drugs, and Acquired Resistance. Cancers.

[68] Delcanale P., Porciani D., Pujals S., Jurkevich A., Chetrusca A., Tawiah K.D., Burke D.H., Albertazzi L. (2020). Aptamers with Tunable Affinity Enable Single-Molecule Tracking and Localization of Membrane Receptors on Living Cancer Cells. Angew. Chem. Int. Ed..

[69] Krause K.M., Serio A.W., Kane T.R., Connolly L.E. (2016). Aminoglycosides: An Overview. Cold Spring Harb. Perspect. Med..

[70] Kim J., Ricci A.J. (2022). In Vivo Real-Time Imaging Reveals Megalin as the Aminoglycoside Gentamicin Transporter into Cochlea Whose Inhibition Is Otoprotective. Proc. Natl. Acad. Sci. USA.

[71] Ma Y., Chiriboga D.E., Pagoto S.L., Rosal M.C., Li W., Merriam P.A., Hébert J.R., Whited M.C., Ockene I.S. (2011). Association between Depression and C-Reactive Protein. Cardiol. Res. Pract..

[72] Bonny A., Tonet J., Márquez M.F., De Sisti A., Temfemo A., Himbert C., Gueffaf F., Larrazet F., Ditah I., Frank R. (2011). C-Reactive Protein Levels in the Brugada Syndrome. Cardiol. Res. Pract..

[73] Yeh E.T.H., Willerson J.T. (2003). Coming of Age of C-Reactive Protein: Using Inflammation Markers in Cardiology. Circulation.

[74] Corti R., Fuster V., Badimon J.J. (2003). Pathogenetic Concepts of Acute Coronary Syndromes. J. Am. Coll. Cardiol..

[75] Harwardt M.-L.I.E., Schröder M.S., Li Y., Malkusch S., Freund P., Gupta S., Janjic N., Strauss S., Jungmann R., Dietz M.S. (2020). Single-Molecule Super-Resolution Microscopy Reveals Heteromeric Complexes of MET and EGFR upon Ligand Activation. Int. J. Mol. Sci..

[76] Catapano C., Rahm J.V., Omer M., Teodori L., Kjems J., Dietz M.S., Heilemann M. (2023). Biased Activation of the Receptor Tyrosine Kinase HER2. Cell. Mol. Life Sci..

[77] Xue T., Feng W., Yu H., Zhu M., Fei M., Bao Y., Wang X., Ma W., Lv G., Guan J. (2017). Metastasis-Associated Protein 1 Is Involved in Angiogenesis after Transarterial Chemoembolization Treatment. BioMed Res. Int..

[78] Miao L., Yan C., Chen Y., Zhou W., Zhou X., Qiao Q., Xu Z. (2023). SIM Imaging Resolves Endocytosis of SARS-CoV-2 Spike RBD in Living Cells. Cell Chem. Biol..

[79] Gray R.D.M., Albrecht D., Beerli C., Huttunen M., Cohen G.H., White I.J., Burden J.J., Henriques R., Mercer J. (2019). Nanoscale Polarization of the Entry Fusion Complex of Vaccinia Virus Drives Efficient Fusion. Nat. Microbiol..

[80] Los G.V., Encell L.P., McDougall M.G., Hartzell D.D., Karassina N., Zimprich C., Wood M.G., Learish R., Ohana R.F., Urh M. (2008). HaloTag: A Novel Protein Labeling Technology for Cell Imaging and Protein Analysis. ACS Chem. Biol..

[81] Holtmannspötter M., Wienbeuker E., Dellmann T., Watrinet I., Garcia-Sáez A.J., Johnsson K., Kurre R., Piehler J. (2023). Reversible Live-Cell Labeling with Retro-engineered HaloTags Enables Long-Term High- and Super-Resolution Imaging. Angew. Chem. Int. Ed..

[82] Von Dassow G., Valley J., Robbins K. (2019). Microinjection of Oocytes and Embryos with Synthetic MRNA Encoding Molecular Probes. Methods in Cell Biology.

[83] Crawford R., Torella J.P., Aigrain L., Plochowietz A., Gryte K., Uphoff S., Kapanidis A.N. (2013). Long-Lived Intracellular Single-Molecule Fluorescence Using Electroporated Molecules. Biophys. J..

[84] Di Paolo D., Afanzar O., Armitage J.P., Berry R.M. (2016). Single-Molecule Imaging of Electroporated Dye-Labelled CheY in Live *Escherichia coli*. Philos. Trans. R. Soc. B Biol. Sci..

[85] Chen Z., Cao Y., Lin C.-W., Alvarez S., Oh D., Yang P., Groves J.T. (2021). Nanopore-Mediated Protein Delivery Enabling Three-Color Single-Molecule Tracking in Living Cells. Proc. Natl. Acad. Sci. USA.

[86] Tsunoyama T.A., Watanabe Y., Goto J., Naito K., Kasai R.S., Suzuki K.G.N., Fujiwara T.K., Kusumi A. (2018). Super-Long Single-Molecule Tracking Reveals Dynamic-Anchorage-Induced Integrin Function. Nat. Chem. Biol..

[87] Basu S., Needham L.-M., Lando D., Taylor E.J.R., Wohlfahrt K.J., Shah D., Boucher W., Tan Y.L., Bates L.E., Tkachenko O. (2018). FRET-Enhanced Photostability Allows Improved Single-Molecule Tracking of Proteins and Protein Complexes in Live Mammalian Cells. Nat. Commun..

[88] Richter V., Lanzerstorfer P., Weghuber J., Schneckenburger H. (2020). Super-Resolution Live Cell Microscopy of Membrane-Proximal Fluorophores. Int. J. Mol. Sci..

[89] Liu Z., Xing D., Su Q.P., Zhu Y., Zhang J., Kong X., Xue B., Wang S., Sun H., Tao Y. (2014). Super-Resolution Imaging and Tracking of Protein–Protein Interactions in Sub-Diffraction Cellular Space. Nat. Commun..

[90] Roudot P., Legant W.R., Zou Q., Dean K.M., Isogai T., Welf E.S., David A.F., Gerlich D.W., Fiolka R., Betzig E. (2020). U-Track 3D: Measuring and Interrogating Dense Particle Dynamics in Three Dimensions. bioRxiv.

[91] Ruthardt N., Lamb D.C., Bräuchle C. (2011). Single-Particle Tracking as a Quantitative Microscopy-Based Approach to Unravel Cell Entry Mechanisms of Viruses and Pharmaceutical Nanoparticles. Mol. Ther..

[92] Von Diezmann L., Shechtman Y., Moerner W.E. (2017). Three-Dimensional Localization of Single Molecules for Super-Resolution Imaging and Single-Particle Tracking. Chem. Rev..

[93] Guo A.-Y., Zhang Y.-M., Wang L., Bai D., Xu Y.-P., Wu W.-Q. (2021). Single-Molecule Imaging in Living Plant Cells: A Methodological Review. Int. J. Mol. Sci..

[94] Gelles J., Schnapp B.J., Sheetz M.P. (1988). Tracking Kinesin-Driven Movements with Nanometre-Scale Precision. Nature.

[95] Koyama-Honda I., Ritchie K., Fujiwara T., Iino R., Murakoshi H., Kasai R.S., Kusumi A. (2005). Fluorescence Imaging for Monitoring the Colocalization of Two Single Molecules in Living Cells. Biophys. J..

[96] Kou L., Jin L., Lei H., Hu C., Li H., Hu X., Hu X. (2019). Real-time Parallel 3D Multiple Particle Tracking with Single Molecule Centrifugal Force Microscopy. J. Microsc..

[97] Small A. (2016). Multifluorophore Localization as a Percolation Problem: Limits to Density and Precision. J. Opt. Soc. Am. A.

[98] Hirsch M., Wareham R., Yoon J.W., Rolfe D.J., Zanetti-Domingues L.C., Hobson M.P., Parker P.J., Martin-Fernandez M.L., Singh S.S. (2019). A Global Sampler of Single Particle Tracking Solutions for Single Molecule Microscopy. PLoS ONE.

[99] Li Y., Shi W., Liu S., Cavka I., Wu Y.-L., Matti U., Wu D., Koehler S., Ries J. (2022). Global Fitting for High-Accuracy Multi-Channel Single-Molecule Localization. Nat. Commun..

[100] Yasui M., Hiroshima M., Kozuka J., Sako Y., Ueda M. (2018). Automated Single-Molecule Imaging in Living Cells. Nat. Commun..

[101] Nixon-Abell J., Obara C.J., Weigel A.V., Li D., Legant W.R., Xu C.S., Pasolli H.A., Harvey K., Hess H.F., Betzig E. (2016). Increased Spatiotemporal Resolution Reveals Highly Dynamic Dense Tubular Matrices in the Peripheral ER. Science.

[102] Chiu S.-W., Leake M.C. (2011). Functioning Nanomachines Seen in Real-Time in Living Bacteria Using Single-Molecule and Super-Resolution Fluorescence Imaging. Int. J. Mol. Sci..

[103] Shaner N.C., Steinbach P.A., Tsien R.Y. (2005). A Guide to Choosing Fluorescent Proteins. Nat. Methods.

[104] Fairlamb M.S., Whitaker A.M., Bain F.E., Spies M., Freudenthal B.D. (2021). Construction of a Three-Color Prism-Based TIRF Microscope to Study the Interactions and Dynamics of Macromolecules. Biology.

[105] Kellermayer M.S.Z., Karsai Á., Kengyel A., Nagy A., Bianco P., Huber T., Kulcsár Á., Niedetzky C., Proksch R., Grama L. (2006). Spatially and Temporally Synchronized Atomic Force and Total Internal Reflection Fluorescence Microscopy for Imaging and Manipulating Cells and Biomolecules. Biophys. J..

[106] Ozadam H., Tonn T., Han C.M., Segura A., Hoskins I., Rao S., Ghatpande V., Tran D., Catoe D., Salit M. (2023). Single-Cell Quantification of Ribosome Occupancy in Early Mouse Development. Nature.

[107] Yafia M., Ymbern O., Olanrewaju A.O., Parandakh A., Sohrabi Kashani A., Renault J., Jin Z., Kim G., Ng A., Juncker D. (2022). Microfluidic Chain Reaction of Structurally Programmed Capillary Flow Events. Nature.

[108] Krainer G., Saar K.L., Arter W.E., Welsh T.J., Czekalska M.A., Jacquat R.P.B., Peter Q., Traberg W.C., Pujari A., Jayaram A.K. (2023). Direct Digital Sensing of Protein Biomarkers in Solution. Nat. Commun..

[109] Nishikawa M., Kanno H., Zhou Y., Xiao T.-H., Suzuki T., Ibayashi Y., Harmon J., Takizawa S., Hiramatsu K., Nitta N. (2021). Massive Image-Based Single-Cell Profiling Reveals High Levels of Circulating Platelet Aggregates in Patients with COVID-19. Nat. Commun..

